# Etiology and Outcomes of Hypernatremia in a Tertiary Pediatric Intensive Care Unit: The Role of Disease Severity

**DOI:** 10.3390/children13091192

**Published:** 2026-09-04

**Authors:** Özlem Yüksel Aksoy, Mustafa Orhan Duyar, Serhat Kaya, Binnaz Çelik, Funda Baştuğ, Murat Doğan, Adem Dursun, Serkan Özsoylu

**Affiliations:** 1 Department of Pediatric Nephrology, Başkent University, 06490 Ankara, Türkiye; 2 Department of Pediatrics, University of Health Sciences, Kayseri City Hospital, 38080 Kayseri, Türkiye; 3 Department of Pediatric Nephrology, Kayseri City Hospital, 38080 Kayseri, Türkiye; 4 Department of Pediatric Emergency, University of Health Sciences, Kayseri City Hospital, 38080 Kayseri, Türkiye; 5 Pediatric Intensive Care Unit, Department of Pediatrics, Liv Hospital, 34340 Istanbul, Türkiye; 6 Pediatric Intensive Care Unit, Department of Pediatrics, Kutahya University of Health Sciences, Kutahya City Hospital, 43100 Kutahya, Türkiye

**Keywords:** hypernatremia, pediatric intensive care unit, critically ill children

## Abstract

**Highlights:**

**What are the main findings?**

- Hypernatremia in critically ill children was associated with a high in-hospital mortality rate (15.9%), with mortality varying according to the underlying disease category.
- Illness severity, reflected by the PRISM score, was the only independent predictor of mortality, whereas the severity and identified etiology of hypernatremia were not independently associated with mortality.

**What are the implications of the main findings?**

- Hypernatremia may serve as a marker of severe underlying illness and complex fluid and electrolyte disturbances rather than an independent determinant of mortality in critically ill children.
- Careful, gradual correction of hypernatremia remains important, while clinical management should also address the underlying disease and overall severity of illness.

**Abstract:**

**Purpose:** Hypernatremia is a common electrolyte disorder in pediatric intensive care units (PICU) and is associated with considerable morbidity and mortality. We aimed to evaluate underlying etiologies, clinical characteristics, and factors associated with outcomes in critically ill children with hypernatremia, while accounting for illness severity. **Methods:** We retrospectively analyzed pediatric patients with at least one serum sodium measurement > 145 mEq/L during their stay in a tertiary Level 3 PICU. Demographic, clinical, laboratory, Glasgow Coma Scale (GCS), and Pediatric Risk of Mortality (PRISM) data were collected. Factors associated with in-hospital mortality were evaluated using univariate and multivariable analyses. **Results:** A total of 113 children were included (mean age 4.8 ± 5.3 years; 57.5% male). Neurological disorders were the most common underlying disease category, while free water deficit was the most frequently identified etiology of hypernatremia. Overall in-hospital mortality was 15.9%. Mortality rates were 21.5% among patients with PICU-associated hypernatremia and 8.3% among those with hypernatremia within the first 24 h of admission; the difference was not statistically significant (*p* = 0.058). PRISM score remained independently associated with mortality (OR 1.124, 95% CI 1.059–1.193; *p* < 0.001), whereas serum sodium concentration was not significantly different between survivors and non-survivors. **Conclusions:** Among critically ill children with hypernatremia, mortality was higher than the overall PICU mortality rate but was primarily associated with overall illness severity rather than with the degree of hypernatremia itself. PRISM score, rather than serum sodium concentration, was independently associated with mortality, suggesting that hypernatremia may represent a marker of severe underlying illness rather than an independent determinant of outcome.

## 1. Introduction

Hypernatremia is a common electrolyte disturbance in pediatric intensive care units (PICUs) and is associated with significant morbidity and adverse clinical outcomes [1,2]. It typically results from a relative deficit of free water and may arise in a wide range of clinical conditions, including dehydration, infections, renal dysfunction, and iatrogenic fluid imbalances [3,4,5]. In critically ill children, impaired thirst response, limited access to free water, and ongoing insensible or gastrointestinal losses further predispose to the development of hypernatremia [6]. Management requires careful restoration of water balance and close monitoring of serum sodium concentrations, as excessively rapid correction may precipitate cerebral edema and other neurological complications, particularly in chronic hypernatremia [7].

Previous studies in different clinical populations have suggested an association between hypernatremia and adverse outcomes. In acutely ill pediatric patients, moderate-to-severe hypernatremia has been associated with an increased risk of death [8]. Likewise, studies in adult intensive care unit (ICU) patients with ICU-acquired hypernatremia have reported higher in-hospital mortality [9]. However, whether hypernatremia itself directly contributes to poor outcomes or rather reflects the severity of the underlying disease remains a matter of debate [10]. In addition, conditions such as acute kidney injury and multiorgan dysfunction may coexist with hypernatremia and confound its relationship with mortality. Consistent with this concept, hypernatremia in adults with acute kidney injury has been associated with an increased risk of major adverse kidney events and mortality [11].

Therefore, in this study, we aimed to evaluate the etiologies, clinical characteristics, and outcomes of hypernatremia in a tertiary PICU cohort, with a particular focus on the relationship between underlying disease processes and clinical outcomes. In addition, we sought to examine the association between hypernatremia and mortality after accounting for illness severity using established clinical severity measures, including the Pediatric Risk of Mortality (PRISM) score and the Glasgow Coma Scale (GCS).

## 2. Materials and Methods

### 2.1. Study Design and Population

This retrospective study was conducted in the tertiary (Level 3) pediatric intensive care unit (PICU) of Kayseri City Hospital, Türkiye. Pediatric patients hospitalized between June 2018 and December 2020 were screened. Patients with at least one serum sodium measurement > 145 mEq/L during their Level 3 PICU stay were considered eligible for inclusion. The Level 3 PICU at the institution predominantly admits critically ill children referred from a wide geographical region. During the study period, the overall annual mortality rate of the unit was approximately 8%, reflecting the high-acuity case mix [12].

Hypernatremia was managed according to institutional practice, with treatment individualized according to the underlying etiology, volume status, and clinical condition. The primary therapeutic goal was gradual correction of serum sodium while avoiding excessively rapid decreases because of the risk of cerebral edema. In general, a decline in serum sodium of no more than 0.5 mEq/L per hour (approximately 10–12 mEq/L per day) was targeted in patients with chronic or unknown-duration hypernatremia, with serial monitoring and adjustment of fluid therapy as clinically indicated.

Clinical, laboratory, and outcome data were obtained from the hospital’s electronic medical record system. The retrospective review was based on electronically documented clinical information and laboratory results.

Initially, 196 patients met the inclusion criteria. However, patients admitted primarily following respiratory or cardiac arrest, or those who experienced respiratory or cardiac arrest before the development of hypernatremia, were excluded from the final analysis because these conditions represent distinct clinical scenarios with inherently high mortality that could confound the relationship between hypernatremia and clinical outcomes. In addition, patients with prolonged PICU stays exceeding two months were excluded because their extended hospitalization could disproportionately influence clinical outcomes and length-of-stay analyses. After these exclusions, a total of 113 patients were included in the final study cohort. Only three patients had multiple PICU admissions during the study period. To avoid repeated observations from the same patient, only one hospitalization per patient was included in the analysis. In these cases, the first eligible admission was selected.

### 2.2. Definitions

Hypernatremia was defined as a serum sodium level > 145 mEq/L [11,13,14]. Patients were further categorized according to the severity of hypernatremia as mild (146–149 mEq/L), moderate (150–159 mEq/L), and severe (≥160 mEq/L) [15,16]. Resolution of hypernatremia was defined as a decrease in serum sodium level to ≤145 mEq/L.

The potential mechanisms underlying hypernatremia were retrospectively reassessed using the available clinical and laboratory data. Based on the presumed pathophysiological mechanism, hypernatremia was classified into five categories: free-water deficit, renal water loss, sodium excess, mixed/multifactorial mechanisms, and unidentified etiology. Free-water deficit included documented dehydration, gastrointestinal fluid losses, inadequate oral or enteral fluid intake, fever or increased insensible losses, and other causes of free-water loss. Renal water loss included diabetes insipidus, osmotic diuresis, diuretic-associated water loss, and other documented renal water losses. Sodium excess included inappropriate administration of sodium-containing fluids, hypertonic fluids, sodium bicarbonate, or other documented sodium loads. Cases with more than one plausible contributing mechanism were classified as mixed/multifactorial. When no specific mechanism could be reliably established from the available records, the etiology was classified as unidentified.

Underlying disease categories were grouped as neurological/central, systemic infection, gastrointestinal disorders (including acute gastroenteritis), renal, cardiac, and others (including intoxication, trauma, and metabolic disorders). Systemic infections included sepsis and severe lower respiratory tract infections such as pneumonia and bronchiolitis requiring intensive care. Patients with acute gastroenteritis or predominant gastrointestinal fluid losses (e.g., vomiting and/or diarrhea) were classified under the gastrointestinal disease category.

PRISM score and GCS at PICU admission were used as indicators of illness severity and neurological status, respectively. Patients were also categorized into two groups according to the timing of hypernatremia onset: those who were hypernatremic within the first 24 h of PICU admission and those who developed hypernatremia more than 24 h after PICU admission. The latter group was considered to have PICU-associated hypernatremia.

### 2.3. Data Collection

Demographic characteristics (age, sex), underlying disease categories, etiology and timing of hypernatremia, laboratory parameters (serum sodium, blood urea nitrogen, creatinine, potassium, chloride, and uric acid), GCS at PICU admission, PRISM score, length of PICU stay, and time to resolution of hypernatremia were recorded.

### 2.4. Outcome Measures

The primary outcome of the study was in-hospital mortality. Secondary outcomes included length of PICU stay and time to resolution of hypernatremia.

### 2.5. Statistical Analysis

Statistical analyses were performed using IBM SPSS Statistics software version 20. Continuous variables were expressed as mean ± standard deviation or median (interquartile range), depending on distribution. Categorical variables were presented as frequencies and percentages.

Comparisons between groups were performed using the independent-samples *t* test or the Mann–Whitney U test for continuous variables, as appropriate, and the chi-square test for categorical variables. Correlations between continuous variables were evaluated using Pearson or Spearman correlation analysis, according to the distribution of the variables.

Clinically relevant variables, including age, serum sodium level, serum creatinine, and PRISM score, were entered into a multivariate logistic regression model to identify independent predictors of in-hospital mortality. Odds ratios (ORs) and 95% confidence intervals (95% CIs) were calculated. A two-sided *p* value < 0.05 was considered statistically significant.

### 2.6. Ethical Approval

The study was approved by the Ethics Committee of Kayseri City Hospital (Date: 18 March 2021, Decision No: 330). The study was conducted in accordance with the principles of the Declaration of Helsinki. Informed consent was waived due to the retrospective nature of the study in accordance with ethical guidelines.

## 3. Results

### 3.1. Patient Characteristics

A total of 113 pediatric patients who had hypernatremia either within the first 24 h of PICU admission or later during their PICU stay met the inclusion criteria and were included in the final analysis. The mean age was 4.8 ± 5.3 years (median 1.9 years; range 1 month–17.5 years), and 65 patients (57.5%) were male. Neurological disorders were the most common underlying disease category (31.0%), followed by miscellaneous conditions including trauma, intoxication, and metabolic diseases (25.7%), gastrointestinal disorders (including acute gastroenteritis) (19.5%), renal diseases (10.6%), systemic infections (9.7%), and cardiac diseases (3.5%).

The median Glasgow Coma Scale (GCS) score at PICU admission was 11 (IQR, 7–14), and the median PRISM score was 9 (IQR, 5–16). Baseline demographic and clinical characteristics are summarized in Table 1.

### 3.2. Characteristics of Hypernatremia

Serum sodium concentrations ranged from 146 to 188 mEq/L (mean 151.3 ± 7.5 mEq/L). Hypernatremia was classified as mild (146–149 mEq/L) in 62 patients (54.9%), moderate (150–159 mEq/L) in 41 (36.3%), and severe (≥160 mEq/L) in 10 (8.8%). Free water deficit was the most frequently identified etiology of hypernatremia (38.9%), followed by renal water loss (17.7%), mixed/multifactorial causes (15.0%), sodium excess (12.4%), and unidentified causes (15.9%). Among patients classified in the sodium excess group, 10 patients were found to develop hypernatremia during treatment with hypertonic/hypernatremic fluids administered for the management of cerebral edema. In addition, one patient was documented to develop hypernatremia following sodium valproate intoxication.

Of the 113 patients, 48 (42.5%) were hypernatremic within the first 24 h of PICU admission, whereas 65 (57.5%) developed hypernatremia more than 24 h after admission and were classified as having PICU-associated hypernatremia. There were no significant differences between the two groups in terms of PRISM score (*p* = 0.677), age (*p* = 0.623), or Glasgow Coma Scale score (*p* = 0.236).

The mean time to normalization of serum sodium was 2.7 ± 2.4 days. The mean PICU length of stay was 17.7 ± 13.3 days.

### 3.3. Mortality

Overall in-hospital mortality was 15.9% (18/113). During the study period, the overall annual mortality rate in the tertiary Level 3 PICU was approximately 8% [12]. Thus, the mortality observed in the present cohort of children with hypernatremia was descriptively higher than the overall PICU mortality. Mortality differed significantly according to the underlying disease category (Pearson χ^2^ = 12.50, *p* = 0.029; Monte Carlo *p* = 0.030). The highest mortality was observed among patients with miscellaneous conditions, including trauma, intoxication, and metabolic disorders (34.5%), followed by renal diseases (16.7%) and neurological disorders (14.3%).

Mortality rates varied according to the etiology of hypernatremia, ranging from 10.0% in patients with renal water loss to 35.3% in those with mixed etiologies; however, this difference was not statistically significant (Pearson χ^2^ = 6.60, *p* = 0.159; Monte Carlo *p* = 0.155).

Compared with survivors, non-survivors had significantly higher PRISM scores (Mann–Whitney U = 358.5, *p* < 0.001) and lower Glasgow Coma Scale (GCS) scores (Mann–Whitney U = 582.5, *p* = 0.032), indicating greater illness severity at PICU admission.

Mortality rates were 21.5% among patients with PICU-associated hypernatremia and 8.3% among those who were hypernatremic within the first 24 h of PICU admission; however, this difference was not statistically significant (Pearson chi-square test, *p* = 0.058).

### 3.4. Univariate and Correlation Analyses

No significant differences were observed between survivors and non-survivors regarding age, serum sodium, blood urea nitrogen, serum creatinine, potassium, chloride, length of PICU stay, or time to normalization of hypernatremia (all *p* > 0.05) (Table 2). Uric acid levels were lower among non-survivors, although this association reached only borderline statistical significance in the parametric analysis.

Spearman correlation analysis demonstrated a moderate positive correlation between serum sodium concentration and blood urea nitrogen (ρ = 0.430, *p* < 0.001) and a weak positive correlation between serum sodium concentration and serum creatinine (ρ = 0.213, *p* = 0.023).

PRISM score showed a significant positive correlation with in-hospital mortality (ρ = 0.369, *p* < 0.001). In contrast, serum sodium concentration was not significantly correlated with either PRISM score (ρ = −0.016, *p* = 0.862) or in-hospital mortality (ρ = 0.058, *p* = 0.540).

Among survivors, serum sodium concentration showed a weak inverse correlation with PICU length of stay (ρ = −0.203, *p* = 0.049). Time to normalization of serum sodium demonstrated a weak positive correlation with PICU length of stay (ρ = 0.221, *p* = 0.041), whereas no significant association was observed between sodium correction time and PRISM score. Acid–base status was not associated with in-hospital mortality (Fisher’s exact test with Monte Carlo simulation, *p* = 0.446).

### 3.5. Multivariate Analysis

A multivariable logistic regression model including age, PRISM score, serum sodium concentration, and serum creatinine was performed to identify independent predictors of in-hospital mortality. The model was statistically significant (Omnibus χ^2^ = 20.35, *p* < 0.001), demonstrated good calibration (Hosmer–Lemeshow *p* = 0.620), and explained 28.2% of the variance in mortality (Nagelkerke R^2^ = 0.282).

Among the variables included in the model, only the PRISM score was independently associated with in-hospital mortality (OR 1.124, 95% CI 1.059–1.193; *p* < 0.001). Age, serum sodium concentration, and serum creatinine were not independently associated with mortality (Table 3).

## 4. Discussion

Hypernatremia is a common electrolyte disturbance encountered in critically ill children and is associated with adverse clinical outcomes. In the present study, we evaluated its etiologies, clinical characteristics, and outcomes in children admitted to a tertiary Level 3 PICU population and demonstrated several clinically relevant findings.

First, the overall in-hospital mortality rate in our cohort was 15.9%, which was higher than the approximately 8% annual mortality reported for the overall Level 3 PICU population at the institution during the study period [12]. This finding suggests that children with hypernatremia represented a particularly high-risk subgroup. However, it should be noted that the Level 3 PICU predominantly admits children with higher acuity and more complex conditions, which may partly explain the mortality observed. Previous studies have consistently reported an association between hypernatremia and increased mortality in critically ill patients [13,17]. In the present study, however, hypernatremia severity was not independently associated with mortality.

Despite not reaching statistical significance, a clinically notable difference in mortality was observed in our study according to the timing of hypernatremia onset. Patients who developed hypernatremia more than 24 h after PICU admission had a higher mortality rate than those who were already hypernatremic within the first 24 h (21.5% vs. 8.3%, *p* = 0.058). This finding may suggest that hypernatremia developing during the PICU stay reflects the severity and complexity of the underlying critical illness and its subsequent management rather than representing an isolated electrolyte abnormality. In contrast, hypernatremia present early after admission may more often reflect the condition that led to PICU admission, including pre-existing free water deficits.

The identified etiology of hypernatremia was not significantly associated with mortality (Pearson χ^2^ = 6.60, *p* = 0.159; Monte Carlo *p* = 0.155). Nevertheless, mortality was descriptively highest among patients with mixed/multifactorial etiologies (35.3%), followed by those with sodium excess (21.4%). The latter group included patients who received hypernatremic or hypertonic fluids for the management of cerebral edema, indicating that hypernatremia in some critically ill children may occur as part of the therapeutic management of severe neurological disease. Therefore, the clinical context and timing of hypernatremia should be considered when interpreting its association with outcomes.

In critically ill children, disturbances in sodium balance may also reflect the challenges of fluid and electrolyte management in addition to the severity of the underlying disease. Rather, after adjustment for illness severity, as assessed by the PRISM score, the PRISM score remained independently associated with mortality, whereas serum sodium concentration and the identified etiology of hypernatremia did not show an independent association with mortality. These findings suggest that hypernatremia may primarily represent a marker of underlying critical illness rather than an independent determinant of outcome. In addition, disturbances in sodium balance may reflect the challenges of fluid and electrolyte management in critically ill children, in addition to disease severity. Mortality differed according to underlying disease category in univariate analysis, with the highest mortality observed among patients with miscellaneous conditions, including intoxication, trauma, metabolic disorders, and other less common diagnoses. However, because of the limited number of patients in several diagnostic subgroups, a stable multivariable model including disease category could not be obtained.

The relationship between hypernatremia and adverse outcomes may be particularly relevant in children with renal dysfunction. Previous studies have similarly reported worse outcomes among patients with acute kidney injury (AKI) and concomitant hypernatremia [11,18]. Hypernatremia in the setting of AKI has been associated with increased mortality, greater decline in renal function, and higher dialysis requirements, while higher serum sodium levels at AKI onset have been linked to increased in-hospital mortality. Taken together, these findings suggest that sodium disturbances may reflect the severity of systemic illness and renal dysfunction rather than represent an isolated therapeutic target.

Dehydration is one of the leading causes of hypernatremia and reflects a deficit in free water rather than sodium excess. It is particularly common in patients with impaired access to water or altered thirst mechanisms and may be exacerbated by conditions such as volume depletion or diabetes insipidus. Management primarily focuses on careful restoration of water balance, typically with hypotonic fluids, while avoiding rapid correction that may lead to neurological complications. Despite established treatment principles, managing dehydration-associated hypernatremia remains challenging, especially in critically ill patients, where both overly rapid and insufficient correction have been linked to adverse outcomes [6]. Also, the increasing population of medically complex children, particularly those with severe disabilities who are unable to maintain adequate hydration due to impaired thirst perception or limited access to free water, may contribute to the development of hypernatremia [4,7,19,20]. Neurological disorders constituted the largest underlying disease category in our cohort. Reduced consciousness, impaired thirst, and dependence on caregivers may predispose these patients to free-water deficit.

As expected, PRISM scores were significantly higher among non-survivors and remained independently associated with mortality in the multivariable analysis. Given that PRISM is a well-validated measure of illness severity in critically ill children [21], this finding supports the interpretation that overall illness severity, rather than serum sodium concentration alone, was more strongly associated with clinical outcome in our cohort. Although GCS was significantly lower among non-survivors in univariate analysis, it was not included in the final multivariable model because PRISM already incorporates neurological assessment and provides a more comprehensive measure of illness severity.

The prognostic significance of serum sodium concentration has also been demonstrated in other critically ill populations. Previous studies, including studies of patients with severe traumatic brain injury, have shown that both elevated sodium levels at ICU admission and fluctuations in sodium levels during hospitalization are associated with worse clinical outcomes [22,23]. Higher sodium levels have been linked to increased mortality, longer hospital and ICU stay, and prolonged mechanical ventilation [23,24]. In addition, sodium levels were found to correlate with established severity scores, suggesting that sodium disturbances may reflect overall illness severity. Notably, variability in sodium levels over time appeared to be independently associated with mortality, regardless of absolute sodium values. These findings support the notion that disturbances in sodium balance may act more as markers of disease severity and physiological instability rather than isolated determinants of outcome.

This study has several limitations. First, the retrospective and single-center design limits the generalizability of our findings and precludes definitive conclusions regarding causality. Second, the retrospective nature of the study limited the precise determination of the underlying mechanisms of hypernatremia in all patients, resulting in a proportion of cases with unidentified etiology. Although PRISM and GCS were available for analysis, the retrospective dataset did not contain complete information on additional physiological variables and detailed urine output measurements for all patients. Furthermore, the small number of patients in some etiologic subgroups may have limited the statistical power to detect differences between these groups. In addition, we evaluated the peak serum sodium concentration rather than serial sodium measurements or sodium variability over time. Therefore, we could not assess the temporal relationship between changes in serum sodium and clinical outcomes. Prospective studies incorporating longitudinal sodium trajectories may provide further insight into the prognostic significance of hypernatremia.

Despite these limitations, our findings suggest that, in pediatric intensive care practice, hypernatremia may represent a marker of illness severity and underlying pathophysiology. Careful correction of serum sodium remains essential; in addition, management should focus on early recognition and treatment of the underlying disease process while appropriately addressing electrolyte disturbances.

## 5. Conclusions

Hypernatremia in critically ill children was associated with substantial mortality; however, serum sodium concentration was not independently associated with in-hospital mortality after adjustment for illness severity. Instead, illness severity, as reflected by the PRISM score, was the only independent predictor of mortality. These findings suggest that hypernatremia may serve primarily as a marker of severe underlying illness rather than an independent determinant of prognosis. Further prospective multicenter studies are needed to better define the relationship between hypernatremia, illness severity, and clinical outcomes in critically ill children.

## Figures and Tables

**Table 1:** Baseline characteristics of the study population (*n* = 113).

Variable	
**Sex, *n* (%)**	
– Male sex, *n* (%)	65 (57.5)
– Female sex, *n* (%)	48 (42.5)
**Age (years), mean ± SD**	4.8 ± 5.3
**PRISM score,** median (Q1–Q3)	9 (5–16)
**Glasgow Coma Scale,** median (Q1–Q3)	11 (7–14)
**Primary disease category, *n* (%)**	
– Neurological/central, *n* (%)	35 (31.0)
– Systemic infection, *n* (%)	11 (9.7)
– Gastrointestinal disorders, *n* (%)	22 (19.5)
– Renal, *n* (%)	12 (10.6)
– Cardiac, *n* (%)	4 (3.5)
– Other, *n* (%)	29 (25.7)
**Etiology of hypernatremia, *n* (%)**	
– Free water deficit, *n* (%)	44 (38.9)
– Renal water loss, *n* (%)	20 (17.7)
– Sodium excess, *n* (%)	14 (12.4)
– Mixed/multifactorial, *n* (%)	17 (15.0)
– Unidentified, *n* (%)	18 (15.9)
**Hypernatremia severity, *n* (%)**	
– Mild (146–149 mEq/L), *n* (%)	62 (54.9)
– Moderate (150–159 mEq/L) *n* (%)	41 (36.3)
– Severe (≥160 mEq/L), *n* (%)	10 (8.8)
**Laboratory findings (mean ± SD)**	
– Serum sodium (range: 135–145 mEq/L)	151.33 ± 7.49
– Serum creatinine (mg/dL) (range: age-dependent)	0.93 ± 1.08
– BUN (range: 7–20 mg/dL)	23.53 ± 21.85
– Serum potassium (range: 3.5–5.5 mEq/L)	3.95 ± 0.99
– Serum chloride (range: 98–107 mEq/L)	116.25 ± 10.73
– Serum uric acid (mg/dL) (range: age-dependent)	6.8 ± 4.9
Acid–base status, *n* (%) (available in 110 patients)	
Normal	37 (33.6)
Metabolic acidosis	44 (40.0)
Metabolic alkalosis	10 (9.1)
Respiratory acidosis	14 (12.7)
Respiratory alkalosis	5 (4.5)
**Clinical outcomes**	
– Length of PICU stay (days), mean ± SD	17.7 ± 13.3
– Sodium correction time (days), mean ± SD	2.7 ± 2.4
– Mortality, *n* (%)	18 (15.9)

**Table 2:** Comparison of clinical and laboratory characteristics between survivors and non-survivors.

Variable	Survivors (*n* = 95)	Non-Survivors (*n* = 18)	*p* Value *
Age, years, median (Q1–Q3)	2.0 (0.92–8.0)	1.42 (0.77–9.25)	0.959
BUN, mg/dL, median (Q1–Q3)	16.0 (7.0–35.0)	14.5 (7.75–29.75)	0.672
Creatinine, mg/dL, median (Q1–Q3)	0.60 (0.35–1.14)	0.66 (0.30–0.92)	0.841
Sodium, mEq/L, median (Q1–Q3)	149 (147–152)	149.5 (147–153.2)	0.538
Potassium, mEq/L, median (Q1–Q3)	4.0 (3.4–4.4)	3.6 (2.9–4.45)	0.280
Chloride, mEq/L, median (Q1–Q3)	115 (109–123)	113.5 (108.75–121)	0.591
Uric acid, mg/dL, median (Q1–Q3)	5.5 (3.5–10.0)	3.35 (2.0–6.88)	0.043
Underlying disease category, *n* (%)			0.030
Neurological/central nervous system diseases	30 (85.7)	5 (14.3)	
Systemic infection	11 (100)	0 (0)	
Gastrointestinal disorders	21 (95.5)	1 (4.5)	
Cardiac	4 (100)	0 (0)	
Renal	10 (83.3)	2 (16.7)	
Other	19 (65.5)	10 (34.5)	
Etiology of hypernatremia, *n* (%)			0.155
Free water deficit	39 (88.6)	5 (11.4)	
Renal water loss	18 (90.0)	2 (10.0)	
Sodium excess	11 (78.6)	3 (21.4)	
Mixed/multifactorial	11 (64.7)	6 (35.3)	
Unidentified	16 (88.9)	2 (11.1)	
Hypernatremia severity, *n* (%)			0.934
Mild	53 (85.5)	9 (14.5)	
Moderate	34 (82.9)	7 (17.1)	
Severe	8 (80.0)	2 (20.0)	
PRISM score, median (Q1–Q3)	9 (5–14)	18 (10–34)	<0.001
Glasgow Coma Scale, median (Q1–Q3)	11 (8–14)	6.5 (3–14)	0.032
Time to normalization of serum sodium, days, median (Q1–Q3)	2 (1–4)	2 (1–3.5)	0.947
Length of PICU stay, days, median (Q1–Q3)	15 (8–23)	14 (4–37.25)	0.768

* Pearson’s chi-square test with Monte Carlo simulation was used for categorical variables, and the Mann–Whitney U test was used for continuous variables.

**Table 3:** Multivariate logistic regression analysis of factors associated with mortality.

Variable	β (B)	SE	OR (95% CI)	*p* Value
Age (years)	0.001	0.058	1.001 (0.894–1.120)	0.991
PRISM score	0.117	0.030	1.124 (1.059–1.193)	<0.001
Serum sodium (mEq/L)	0.014	0.046	1.014 (0.927–1.110)	0.761
Creatinine (mg/dL)	−0.228	0.407	0.796 (0.359–1.767)	0.575

Multivariable logistic regression model including age, PRISM score, serum sodium concentration, and serum creatinine. Model performance: Omnibus test χ^2^ = 20.348, *p* < 0.001; Hosmer–Lemeshow goodness-of-fit test *p* = 0.620; Nagelkerke R^2^ = 0.282.

## Data Availability

Data is available from the corresponding author on reasonable request. The data are not publicly available due to privacy and ethical restrictions.
